# Leader–Employee Congruence in Humor and Innovative Behavior: The Moderating Role of Dynamic Tenure

**DOI:** 10.3389/fpsyg.2021.579551

**Published:** 2021-03-05

**Authors:** Yue Yuan

**Affiliations:** School of Economics and Management, Tsinghua University, Beijing, China

**Keywords:** humor, innovative behavior, leader–employee congruence, dynamic tenure, polynomial regression

## Abstract

Drawing upon the literature on complementary fit theory, the purpose of this study is to examine how the dynamic tenure moderates the relationship between leader–employee congruence/incongruence in humor and employee innovative behavior. Data were collected from 108 leader–employee dyads from information technology companies in China. Polynomial regression combined with the response surface methodology was used to test the hypotheses. Four conclusions were drawn. First, employee innovative behavior was maximized when leaders and employees were incongruent in humor. Second, in the case of incongruence, employees had higher innovative behavior when employees were more humorous than their leaders. Third, in the case of congruence, employees had higher innovative behavior when a leader’s and an employee’s humor matched at high levels. Fourth, dynamic tenure moderated the leader–employee congruence/incongruence effect of humor on employee innovative behavior. This study enhanced theoretical developments by considering the importance of leaders’ congruence with employees in humor for the first time. Additionally, the research results provided better practical guidance for effectively promoting employee innovative behavior.

## Introduction

Because employee innovative behavior is an important source for an organization to maintain competitive advantage (Shin et al., 2017) and obtain organizational success (Chung et al., 2017; Yuan and Woodman, 2020), it is essential to identify factors that increase innovative behavior (Anderson et al., 2004, 2014; Shin et al., 2017; Li et al., 2020). Leader humor, one of the most important dispositional antecedents of innovative behavior, has thus received research attention (Pundt, 2015; Li et al., 2019; Hu and Luo, 2020). From the perspective of behavior view, leader humor in this study refers to the extent to which a leader uses humor with each employee (Avolio et al., 1999; Cooper et al., 2018). It is a discrete social behavior (Robert and Wilbanks, 2012), which means that leaders intentionally create interesting verbal or non-verbal activities to amuse one particular follower (Pundt and Venz, 2017), including spontaneous verbal humor and sharing interesting stories or jokes. As a communication strategy, leader humor is related to employee creativity (Li et al., 2019; Hu and Luo, 2020; Peng et al., 2020), job satisfaction (Robert et al., 2015), job performance (Kim et al., 2016; Tan et al., 2020a), work engagement (Yam et al., 2018; Tan et al., 2020b), organizational citizenship behavior (Cooper et al., 2018), voice behavior (Liu et al., 2020), and feedback-seeking behavior (Karakowsky et al., 2020). Although researchers have started to use a dyadic and relational approach to examine the outcomes of leader humor, such as leader–member exchange (Pundt and Venz, 2017; Cooper et al., 2018; Yam et al., 2018; Liu et al., 2020) or employee’s trust in the supervisor (Neves and Karagonlar, 2020), a critical question remains regarding the role of employee humor in shaping the relationship between leader humor and innovative behavior. Therefore, the purpose of this study is to examine the relationship between leader–employee congruence/incongruence in humor and employee innovative behavior.

The perspectives of both resources and fit provide a theoretical basis for explaining the relationship between leader–employee congruence in humor and innovative behavior. On the one hand, from the perspective of resources, humor can provide cognitive, emotional, and relational resources for employee innovation (Pundt, 2015; Cooper et al., 2018). First, humor constructs cognitive resources for innovation. Incongruity is a cognitive element of humor (Pundt, 2015), and humorous incongruity stimulates new ways of thinking and playing with ideas and leads to unusual associations and new ideas (Holmes, 2007). Second, humor constructs emotional resources for innovation. Humorous stimuli activate brain regions that are associated with laughter (Robert and Wilbanks, 2012) and positive emotions (Goswami et al., 2016). The activated positive emotions broaden their thought–action repertoires (Fredrickson, 2001), thus promoting the emergence of innovative behavior (Madrid et al., 2014). In addition, employees with positive emotions pay more attention to the value and opportunity of innovation, while ignoring the potential risks of innovative implementation (Gorman et al., 2012). Third, humor constructs relational resources for innovation. Humor helps to overcome the hierarchical differences between leaders and employees and build a sense of psychological security (Pundt, 2015). Hence, employees feel free to develop, communicate, and implement their ideas without any fear of negative consequences (Carmeli et al., 2010). Therefore, this study considers the effect of humor on innovative behavior directly from the perspective of resources for the first time.

On the other hand, from the perspective of fit, the complementary fit between leaders and employees is more conducive to employee innovative behavior. According to person–environment (P-E) fit research (Kristof, 1996; Kristof-Brown et al., 2005), supplementary fit focuses on the similarity between the person and other individuals in the environment, whereas complementary fit occurs when one part (person or organization) provides the other part with what they want (Marstand et al., 2017). As for the innovation, intrapersonal variability perspective of creativity (Barron and Harrington, 1981) points out that individuals who hold different or opposing elements within themselves have greater creativity (Barron and Harrington, 1981; Chang et al., 2015). Therefore, for the first time, this study includes leader humor and employee humor into the model at the same time and tests whether complementary fit is better than supplementary fit.

In addition, dynamic tenure defines the boundary conditions for the effect of leader–employee congruence in humor from the perspectives of both resources and fit. The longer dynamic tenure means that leader and employee developed a better social exchange relationship in the long-term communication and cooperation (Li et al., 2019), which is conducive to the accumulation of human capital (Steffens et al., 2014) and psychological capital (Li et al., 2019). However, from the perspective of resource matching (Halbesleben et al., 2014), employees with shorter dynamic tenure are more likely to cherish existing resources and invest it in innovative activities. Therefore, this study suggests that employees with shorter dynamic tenure are more likely to obtain resources from leader–employee humor incongruence and engage in innovative behavior.

Therefore, drawing upon the literature on complementary fit (Kristof, 1996; Kristof-Brown et al., 2005), this study examines the effects of leader–employee congruence/incongruence in humor on employee innovative behavior that was moderated by dynamic tenure. By hypothesizing and testing these relationships, this study makes important theoretical contributions to the literature on humor and innovation, offering a more balanced perspective that recognizes both the strengths and weaknesses of leader humor from the perspective of congruence. First, this study enriched P-E fit theory by proving the positive results of leader–employee incongruence in humor. Previous research on P-E fit theory ignored the problem of complementary fit (Marstand et al., 2017) and resource matching. This study discussed in detail how employees construct and deal with existing resources and put resources into innovative activities. Second, this study included the leader humor and employee humor into the model at the same time so as to better recognize and understand the connotation of humor from the perspective of leader–employee congruence. Previous studies have not really explored the effect of humor from the perspective of interaction between leaders and employees. This study expanded the research on humor with an obvious individual feature from a single-level perspective to a two-level interaction perspective. Third, this study started with the humor fit perspective and discussed in details about the impact of the leader–employee congruence in humor on stimulating the employee innovative behavior, which expanded the antecedents of innovative behavior. Previous studies have rarely explored the common impact of individual and organizational context interaction on innovation (Anderson et al., 2014). In addition, this study also extended the boundary conditions of innovative behavior from the perspective of dynamic tenure. Fourth, this study also boasted great practical significance. The actual management should also take into account the leader–employee congruence in humor, besides the influence of leader humor on employee innovative behavior. Attention should be paid to complementary fit of leaders and employees in humor, thus giving a role to the humor in positively predicting and promoting employee innovative behavior.

## Theories and Hypotheses

### The Four Different Scenarios of Leader–Employee Congruence in Humor

Humor is a way for leaders and employees to express their feelings in the workplace (Mesmer-Magnus et al., 2012). Because leaders have the ability to influence employees’ perceptions of the environment (LePine et al., 2016) and also set the tone for humor expression at work (Cooper et al., 2018), the existing researches on innovation from the perspective of humor mainly focused on leader humor, ignoring the role of employee humor. However, workplace innovation behavior is embedded in the process of interpersonal interaction (Scott and Bruce, 1994), and the consistency of personal characteristics and environmental factors is very important to promote innovative behavior (Choi, 2004). Therefore, leader humor and employee humor are mutually influential and correlated. When discussing the influence of workplace humor on employee innovative behavior, it is necessary to consider both leader humor and employee humor.

According to the level of leader humor and employee humor, this study identified the following four different matching scenarios as shown in Table 1: high–high, low–low, high-low, low–high. The former two fall into the category of congruence and the latter two into that of incongruence. In discovering the impact that leader–employee congruence in humor has on innovative behavior, this study will address the following: first, whether innovative behavior is higher in incongruence scenarios than in scenarios of congruence; second, between the two incongruence scenarios, whether innovative behavior is higher when employees are at a higher level of humor than leaders in comparison to the opposite; and third, between the two congruence scenarios, whether innovative behavior is higher in a high–high one than in a low–low one.

**TABLE 1:** The four different scenarios of (in)congruence in leader–employee humor.

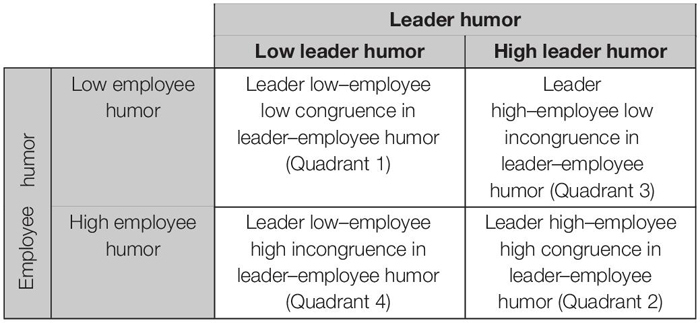

### Humor Congruence Effects on Innovative Behavior

Innovative behavior refers to the generation, communication, and implementation of new ideas concerning products, services, and processes (Pundt, 2015; Shin et al., 2017). Adequate supplies of resources are critical to innovative behavior, such as equipment, facilities, and time (Scott and Bruce, 1994). Resources are defined as anything perceived by the individual to help attain his or her goals (Halbesleben et al., 2014). Humor that makes others happy by sharing interesting events is often associated with positive emotions (Yip and Martin, 2006; Wijewardena et al., 2017; Cooper et al., 2018), which can expand the range of thinking and action of individuals and help to build lasting resources, such as cognitive, social, and psychological resources (Fredrickson, 2001; Cooper et al., 2018). Therefore, humor can help individuals construct cognitive, emotional, and relational resources (Pundt, 2015; Wijewardena et al., 2017; Cooper et al., 2018; Hu and Luo, 2020). The resources that employees apply to innovation mainly come from their own resources and those given by leaders. Therefore, this study intends to explore the influence of leader–employee congruence in humor on innovative behavior from the perspectives of both resources and fit.

The view of complementary fit points out that dissimilarity may have positive consequences for employees (Glomb and Welsh, 2005). For example, based on the dominance complementarity theory, Grant and Berry (2011) proved that leaders rated high in extraversion achieved higher profits when employees were passive. In addition, previous findings supported the intrapersonal variability perspective of creativity (Chang et al., 2015): individuals who hold different or opposing elements within themselves have greater creativity (Barron and Harrington, 1981). Therefore, in the process of interaction between leaders and employees, with humor as an individual resource, when the humor of leaders and employees is complementary fit, employee innovative behavior will be higher.

On the one hand, in the case of incongruence between leader humor and employee humor, they can form a complementary fit in resources so as to construct heterogeneous innovative resources. First, Quadrant 4 (employees are humorous and the leaders are not humorous) means that employees have different innovative resources brought by the humor. At this time, humorous employees are good at using positive emotions to expand thinking flexibility and promote the generation of innovative ideas (Baas et al., 2008). Leaders who are not humorous enough may realize the decrease of leadership effectiveness, which may prompt them to take positive leadership behaviors to improve leadership effectiveness, such as creating an excellent organizational environment for innovation. Second, Quadrant 3 (employees are not humorous and leaders are humorous) means that leaders have different innovative resources brought by humor. At this time, leaders will provide flexible thinking style, positive emotional support, and safe psychological atmosphere to meet the innovative needs of employees (Pundt, 2015; Wijewardena et al., 2017; Li et al., 2019). And employees will cherish the innovative resources and actively participate in innovative activities. Therefore, in the case of incongruence, employees show a more innovative behavior.

On the other hand, in the case of congruence between leader humor and employee humor, they show similar matching in resources, and redundancy or lack of resources will hinder employee innovation. First, Quadrant 2 (both leaders and employees are humorous) means that they have the same innovative resources brought by humor. Although leaders and employees similarity can promote the leader–member exchange quality and thus promote positive work outcomes (Zhang et al., 2012), internal variability perspective of creativity suggested that individuals who hold different or opposing elements within themselves have greater creativity (Barron and Harrington, 1981). In addition, previous studies found that employees who are trusted by leaders may generate more workload and emotional exhaustion (Baer et al., 2015). Second, Quadrant 1 (both leaders and employees are not humorous) means that they have only a few innovative resources brought by humor. Innovation depends on internal and external available information and resources (Scott and Bruce, 1994), and the lack of resources may hinder innovative activities. Therefore, when leaders and employees have the same level of humor, employee innovative behavior is lower.

H1. The higher the incongruence of an employee’s and his or her leader’s levels of humor are, the better the employee innovative behavior.

Leader–employee incongruence in humor has two different situations (Quadrant 3 and Quadrant 4). The essence of innovative behavior is that individuals participate in all stages of innovative activities, including the generation, dissemination, and implementation of ideas (Shin et al., 2017). On the one hand, humor endorser is linked to self-integration (Chang et al., 2015). Self-integration will generate more intrinsic motivation (Weinstein et al., 2013), which is conducive to the generation of innovative behavior (Grant and Berry, 2011; Su et al., 2020). Previous research has found that employees with intrinsic interest in innovation voluntarily engage in innovative behavior, because they naturally prefer and enjoy the engagement in innovative activities (Shin et al., 2017). In addition, humorous employees are also good at using positive emotions to expand thinking flexibility (Baas et al., 2008), and these employees experiencing positive affect are more likely to generate new ideas and stimulate innovative behavior (Mielniczuk and Laguna, 2020). Thus, when employee humor is higher than his or her leader, it means that the employee has stronger intrinsic motivation and more emotional resources for innovative behavior.

On the other hand, if employees lack humor, even if humorous leaders can provide external resources and conditions for employee innovation, it is difficult for employees to apply these resources to innovation, because the important influence factor of innovation is employee’s intrinsic interest in innovation (Shin et al., 2017). For example, previous research has found that extrinsic motivation is positively related to innovation only when the value of rewards is integrated to one’s sense of self (Gupta, 2020). Otherwise, extrinsic motivation is not related to innovation (Gupta, 2020). Thus, when employee humor is lower than his or her leader, it means that the employee has weaker intrinsic motivation and less emotional resources for innovative behavior. Therefore, this study suggests that the intrinsic motivation and emotional resources of employee humor play a more significant role in the pattern of resource allocation and ultimately lead to more innovative behavior.

H2. Innovative behavior is higher when an employee’s humor is higher than a leader’s rather than when a leader’s humor is higher than an employee’s.

While discussing congruence, it needs to be made clear that leaders and employees can be either congruent at a high or low level of humor. On the one hand, both leaders and employees being humorous (Quadrant 2) means that both employees and leaders have a positive emotional experience. Positive emotions expand the scope of cognition and attention (Fredrickson, 2013), which is conducive to the generation and implementation of innovative ideas (Mielniczuk and Laguna, 2020). In addition, the intrinsic motivation brought by humor (Weinstein et al., 2013; Chang et al., 2015) is the internal driving force of innovation (Su et al., 2020). In this case, both leaders and employees are committed to providing motivational and emotional resources for innovation, so employee will produce more innovative behavior.

On the other hand, the lack of humor of both leaders and employees (Quadrant 1) means that employees lack the motivational and emotional resources for innovation, which hinder innovative behavior. If leaders and employees are not humorous, it is difficult for employees to generate intrinsic motivation for innovation (Weinstein et al., 2013; Chang et al., 2015), and it is also difficult for leaders to provide innovative resources for employees. In addition, employees without a sense of humor mean that they cannot feel the positive emotions brought about by their own humor (Baas et al., 2008) and their leader’s humor (Wijewardena et al., 2017), which leads to the lack of emotional resources for innovation. Therefore, compared with the leaders and employees who are not humorous, when both leaders and employees are humorous, employee will be more involved in innovative behavior.

H3. Innovative behavior is higher when an employee is aligned with a leader at a high level of humor rather than when an employee is aligned with a leader at a low level of humor.

### The Moderating Role of Dynamic Tenure

Dynamic tenure refers to the duration of time an employee has worked together with his direct leader in an organization (Li et al., 2019). From the perspective of resources, the longer an individual’s organizational tenure is, the easier it is to obtain human capital (Steffens et al., 2014) and psychological capital (Li et al., 2019), such as job knowledge, skills, abilities, and experiences (Steffens et al., 2014). These resources all contribute to employee innovation. However, empirical evidence showed that the rate of acquiring more tenure-related resources tends to be greater in employees who are in early, rather than advanced, stages of organizational membership (Ng and Feldman, 2013). In other words, when the dynamic tenure is short, employees can get the innovative resources quickly from the organization. Therefore, this study suggests that employees with shorter dynamic tenure are more likely to obtain resources from leader–employee incongruence in humor and engage in innovative behavior.

Employees with shorter dynamic tenure can quickly obtain the innovative resources from the new environment. On the one hand, in terms of the number of resources, employees accumulate more resources and human capital at these early stages of socialization (Steffens et al., 2014). On the other hand, from the perspective of the quality of resources, the initial acquisition of resources is more valuable for employees. Therefore, in the case of leader–employee incongruence in humor, employees with shorter dynamic tenure can put resources into innovative activities more effectively.

By contrary, when the dynamic tenure is long, it is difficult for employees to obtain the innovative resources. On the one hand, a longer dynamic tenure may solidify the thinking style. For example, Woods et al. (2018) found that with the increase of tenure, employees with more conscientiousness showed less innovative behavior. On the other hand, as tenure increases, the relationships between leaders and employees tend to stabilize (Ilies et al., 2005). Innovation needs diversity environment (Chang et al., 2015), and stable leader–member relationship may inhibit innovation. Therefore, it is difficult for employees with longer tenure to engage in innovative activities even when faced with the unique resources brought by the incongruence of leader–employee humor.

**FIGURE 1:**
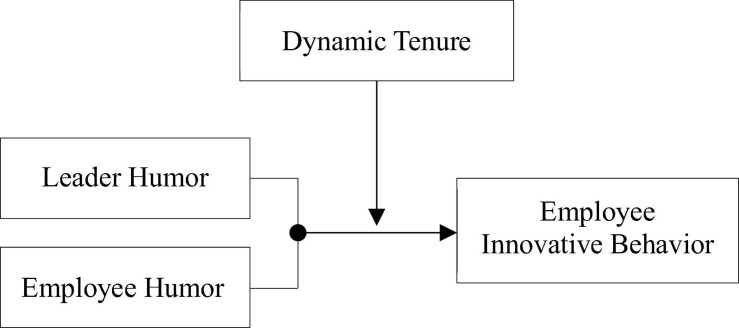
Hypothesized model.

H4. The relationship between leader–employee congruence in humor and innovative behavior will be moderated by dynamic tenure. Specifically, for employees from a low dynamic tenure group, innovative behavior will be positively predicted by increasing incongruence between leader humor and employee humor (Figure 1).

## Materials and Methods

### Participants and Procedures

Data were collected from information technology companies in China, located in Beijing, Chengdu, Guangzhou, and Chongqing. In order to reduce common method variance (Podsakoff et al., 2003), this study adopted multi-time points design. In the first-wave survey (T1), this study sent questionnaires to 300 of employees (response rate of 78.67%). This study surveyed employee demographic variables (e.g., dynamic tenure) and employee humor. And 1 week after the first survey, the second questionnaire (T2) was distributed to the 236 employees and their leaders. This study surveyed the employee innovative behavior and leader humor, and 108 of whom returned complete questionnaires (response rate of 45.76%), constituting the final sample of this study. Among the employees, approximately 51.85% were male, 88.89% had a college degree or better, the average age was 30.21 (*SD* = 6.23) years, and the mean tenure was 6.39 (*SD* = 4.83) years.

### Measures

The language used in this study was Chinese, and this study applied translation/back-translation procedures (Brislin, 1980) to translate the English-based measures into Chinese. All scales were measured using a seven-point Likert format (1 = strongly disagree, 7 = strongly agree).

#### Humor

Leaders and employees assessed their own humor using Avolio et al. (1999) five-item humor instrument. An example item is “I use humor to take the edge off during stressful periods” (leader: α = 0.88, employee: α = 0.87).

#### Innovative Behavior

Employees rated their innovative behavior using the nine-item scale developed by Ng and Lucianetti (2016). An example item is “I transform innovative ideas into useful applications” (α = 0.91).

#### Dynamic Tenure

This continuous variable was measured using self-report responses by employees, who were asked to indicate the amount of time (in years) they had worked for their current direct leader.

#### Control Variables

Previous researches suggested that innovative behavior may be related to demographic characteristics, such as age and education level (Chen et al., 2016). Therefore, this study controlled for employee’s gender, age, education, and tenure.

### Analytic Strategy

In order to test the hypotheses, this study used polynomial regression and response surface methodology (Edwards and Parry, 1993). Specifically, the dependent variable (innovative behavior) was regressed on the control variables, as well as the five polynomial terms, that is, leader humor (LH), employee humor (EH), leader humor squared (LH^2^), leader humor times employee humor (LH ^∗^ EH), and employee humor squared (EH^2^). In other words, this study estimated the following equation (to simplify, all control variables are omitted):

(1) $$I\,n\,n\,o\,v\,a\,t\,i\,v\,e\,b\,e\,h\,a\,v\,i\,o\,r=b_{0}+b_{1}\,L\,H+b_{2}\,E\,H+b_{3}\,L\,H^{2}+\,b_{4}\,L\,H*E\,H+b_{5}\,E\,H^{2}+e$$

As Edwards and Cable (2009) describe, the congruence test involved the slope (b1 + b2) along the congruence line (LH = EH), and the slope (b1 - b2) and curvature (b3 - b4 + b5) along the incongruence line (LH = -EH).

In addition, to test the moderating effect of dynamic tenure on innovative behavior and to directly facilitate comparison of coefficients across the dynamic tenure groups, this study used a Chow test (Chow, 1960). This test is analogous to testing the equality of coefficients in a multisample structural equation model.

## Results

### Confirmatory Factor Analyses

This study conducted confirmatory factor analyses to examine the distinctiveness of the three variables (leader humor, employee humor, and innovative behavior). The results revealed that the three-factor model (χ*^2^* = 271.00, *df* = 149, RMSEA = 0.09, CFI = 0.95, IFI = 0.95, NNFI = 0.94) was superior to all plausible alternative models (Table 2).

**TABLE 2:** Model fit results for confirmatory factor analyses.

Models	χ^2^	*df*	RMSEA	CFI	IFI	NNFI
Three-factor model	271.00	149	0.09	0.95	0.95	0.94
Two-factor model^a^	588.97	151	0.17	0.81	0.81	0.78
One-factor model^b^	996.84	152	0.23	0.63	0.63	0.58

*n = 108. ^*a*^Two-factor model: leader humor + employee humor, innovative behavior. ^*b*^One-factor model: all variables are combined.*

### Correlation Analyses

Table 3 presents the means, standard deviations, and correlations of all study variables. Leader humor (*r* = 0.23, *p* < 0.05) and employee humor (*r* = 0.37, *p* < 0.01) were significantly related to employee innovative behavior.

**TABLE 3:** Descriptive statistics and correlations.

Variables	Mean	SD	1	2	3	4	5	6	7
1.Employee gender	1.48	0.50							
2.Employee age	30.21	6.23	−0.16						
3.Employee education	2.10	0.61	0.05	0.31**					
4.Employee tenure	6.39	4.83	−0.18^+^	0.74**	0.13				
5. Dynamic tenure	2.90	2.98	−0.06	0.54**	0.27**	0.65**			
6. Leader humor	4.19	1.25	−0.05	−0.03	0.03	−0.01	−0.08		
7. Employee humor	4.55	1.03	−0.24*	0.13	−0.003	0.21*	0.18^+^	0.37**	
8. Employee innovative behavior	4.85	1.13	−0.09	−0.03	0.04	0.07	0.16^+^	0.23*	0.37**

n = 108; gender: 1 = male; 2 = female. Age and tenure in years. **p < 0.01, *p < 0.05, ^+^p < 0.1.

### Hypotheses Testing

**FIGURE 2:**
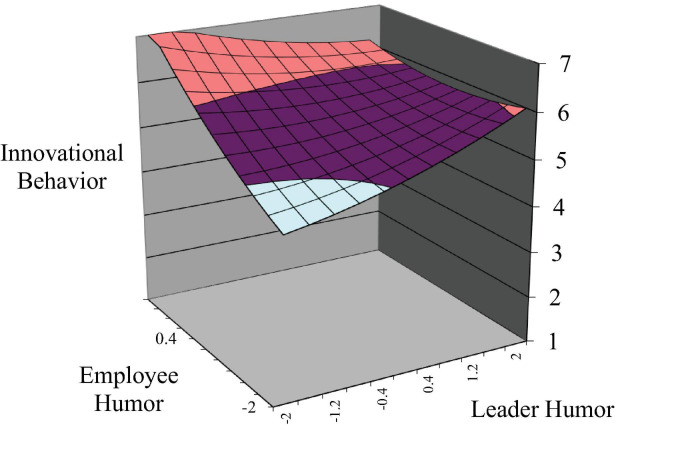
The effect of leader–employee congruence in humor on innovative behavior.

Hypothesis 1 proposed that the higher the incongruence an employee’s and his or her leader’s levels of humor are, the better the employee innovative behavior. As shown in Model 2 of Table 4, the surface along the incongruence line was significantly curved upward (curvature = 0.28, *p* < 0.05), indicating that the incongruence condition has higher innovative behavior than the congruence condition. Furthermore, the results of Monte Carlo analyses revealed that the second principal axis had a slope (p21) that was not significantly different from 1.0 [0.922, 95% confidence interval (CI) = 0.018, 2.225] and an intercept (p20) that was not significantly different from 0 (−1.029, 95% CI = −4.936, 0.012). In order to interpret these results holistically, this study plotted the overall response surface using the coefficient estimates in Figure 2. The concave curvature along the LH = −EH line illustrates that employee innovative behavior increases as leader and employee humor become more discrepant compared to dyads where humor converge. Thus, Hypothesis 1 was supported.

**TABLE 4:** Polynomial regression results of the (in)congruence effects.

	Employee innovative behavior			
	M_1_		M_2_	
	*b*	SE	*b*	SE
Intercept	5.70**	0.77	5.57**	0.73
Employee gender	−0.20	0.22	−0.06	0.22
Employee age	−0.04	0.03	−0.03	0.03
Employee education	0.15	0.19	0.03	0.18
Employee tenure	0.05	0.03	0.03	0.03
Leader humor (LH)			0.08	0.09
Employee humor (EH)			0.36**	0.11
LH^2^			0.05	0.05
LH * EH			−0.17*	0.07
EH^2^			0.06	0.07
*F* value	0.82		3.01**	
*R*^2^	0.03		0.22	
Congruence line (LH = EH)				
Slope (b1 + b2)			0.43**	
Curvature (b3 + b4 + b5)			−0.05	
Incongruence line (LH = −EH)				
Slope (b1 − b2)			−0.28^+^	
Curvature (b3 − b4 + b5)			0.28*	

n = 108; Unstandardized regression coefficients are reported. Gender: 1 = male; 2 = female. **p < 0.01, *p < 0.05, ^+^p < 0.1.

Regarding the asymmetrical incongruence effect (Hypothesis 2), the quantity representing the lateral shift is negative (slope = −0.28, *p* < 0.1), indicating a shift toward the region where employee humor is greater than leader humor. Thus, when an employee’s humor is higher than his or her leader’s, innovative behavior increases more sharply than it does when the employee’s humor is lower than the leader’s, supporting Hypothesis 2. This asymmetrical effect is also shown in Figure 2, in which innovative behavior is higher at the left corner (EH = 2 and LH = −2) than at the right corner (EH = −2 and LH = 2).

Hypothesis 3 suggested that innovative behavior is higher when leader and employee are aligned at a high level of humor as opposed to when they are aligned at a low level. As shown in Table 4, the slope along the congruence line is significant and positive (slope = 0.43, *p* < 0.01), indicating that the high–high congruence condition has higher innovative behavior than the low–low congruence condition. The response surface in Figure 2 also indicates that innovative behavior is higher at the rear corner (high/high congruence) than at the front corner (low/low congruence); thus, Hypothesis 3 was supported.

Turning to Hypothesis 4, which stated that dynamic tenure moderated the relationship between leader–employee congruence in humor and innovative behavior, this study reports the results in Table 5. This study divided the samples according to the median of moderate variable and carried out structural equation analysis in the samples above and below the median, respectively, and compared the differences of coefficients (Lee and Antonakis, 2014). As shown in Table 5, in the low dynamic tenure group, the surface along the incongruence line was significantly curved upward (curvature = 0.39, *p* < 0.05), and the surface along the congruence line was significantly curved downward (curvature = −0.42, *p* < 0.01). However, in the high dynamic tenure group, this trend has become less obvious.

**TABLE 5:** Polynomial regression results of the moderating effects.

Variables	Employee innovative behavior			

	Low dynamic tenure		High dynamic tenure	
	*b*	SE	*b*	SE
Intercept	6.17**	0.97	5.98**	1.39
Employee gender	−0.14	0.31	−0.37	0.31
Employee age	−0.04	0.03	−0.02	0.06
Employee education	0.01	0.29	−0.04	0.24
Employee tenure	−0.04	0.05	0.02	0.07
Leader humor (LH)	−0.05	0.12	0.04	0.16
Employee humor (EH)	0.46**	0.14	0.35	0.21
LH^2^	0.03	0.06	0.16	0.11
LH * EH	−0.40**	0.10	−0.02	0.12
EH^2^	−0.04	0.10	−0.02	0.12
*F* value	3.84**		1.47	
*R*^2^	0.44		0.23	
Congruence line (LH = EH)				
Slope (b1 + b2)	0.41*		0.39*	
Curvature (b3 + b4 + b5)	−0.42**		0.12	
Incongruence line (LH = −EH)				
Slope (b1 − b2)	0.51*		−0.30	
Curvature (b3 − b4 + b5)	0.39*		0.15	
Comparison of the regression coefficients	*df* = 5			
(LH, EH, LH^2^, LH * EH, EH^2^ as IV, IB as DV)	χ = 24.54**			

n = 108; Unstandardized regression coefficients are reported. IB, innovative behavior; IV, independent variable; DV, dependent variable. Gender: 1 = male; 2 = female. **p < 0.01, *p < 0.05.

Next, this study examined whether the five polynomial terms differed across the groups; using Stata’s SUEST command, this study simultaneously tested the difference in regression coefficients b1, b2, b3, b4, and b5 (for the measures LH, EH, LH^2^, LH ^∗^ EH, EH^2^ in Eq. 1), respectively, across the two groups. Results indicated that the coefficients were significantly different [χ^2^(5) = 24.54, *p* < 0.01]. Thus, Hypothesis 4 was supported.

## Discussion

From the dyadic perspective of leaders and employees, this study examined the effect of leader–employee congruence/incongruence in humor on employee innovative behavior and the role of dynamic tenure in moderating such effects. It found that innovative behavior was higher when leaders and employees were incongruent at humor than otherwise. The high–high congruence condition had higher innovative behavior than the low–low congruence condition, and more favorable effects on innovative behavior were produced when employees were at a higher level of humor than leaders. In addition, dynamic tenure moderated the effect of leader–employee congruence/incongruence in humor on employee innovative behavior.

### Theoretical Implications

First, this study enriched P-E fit theory by proving the positive results of leader–employee incongruence in humor. The previous research hypothesizing that similarity between individuals results in positive work outcomes (Zhang et al., 2012), and the role of complementary fit for leadership dynamics remains unexplored (Marstand et al., 2017). Building on this, this study aimed at introducing the complementary fit approach in the innovation field and examined whether dissimilarity results in positive outcomes (innovative behavior) from the perspective of resource matching. This study pointed out that innovative behavior was higher when leaders and employees were incongruent at humor than otherwise. These results emphasized the importance of heterogeneous resources for innovation. Therefore, this study enriched P-E fit theory from the perspective of resources by focusing on complementary fit and resources matching.

Second, this study included the leader humor and employee humor into the model at the same time so as to better recognize and understand the connotation of humor from the perspective of fit. Previous studies explored leader humor from trait view (Yam et al., 2018) and behavior view (Cooper et al., 2018), ignoring the process view. Although Cooper et al. (2018) believed that leader humor fostered high-quality leader-member exchange and, in turn, organizational citizenship behavior, there is a lack of research on exploring related issues from the perspective of interaction between leaders and employees. In addition, previous studies on the effect of leader humor on work outcomes ignored the role of employee humor (Li et al., 2019). Whether the influence of leader humor is positive or negative depends on the personal characteristics of employees (Wood et al., 2011). The results of this study found that employee innovative behavior will be higher, only when the humor of leaders and employees is complementary fit. Therefore, this study described the internal process of leader humor influencing employee innovative behavior from the perspective of interaction process between leader and employee (process view) and enriched the related research on leader humor from the perspective of dual interaction.

Third, this study expanded the antecedents of innovative behavior by discussing in details about the impact of the humor congruence on stimulating the employee innovative behavior. On the one hand, previous study neglected the role of employee humor in exploring the impact of leader humor on innovative behavior (Li et al., 2019). Leader humor and employee humor may interact to influence work outcomes. For example, leaders’ self-defeating humor was positively associated with LMX when followers were high in self-defeating humor (Wisse and Rietzschel, 2014). On the other hand, previous studies explored the influencing factors of innovation from the individual level, such as traits, values, thinking styles, self-concepts and identity, knowledge and abilities, and psychological states (Anderson et al., 2014), and neglected the perspective of resources. Innovation depends on internal and external available resources (Scott and Bruce, 1994). Therefore, this study found that in the case of leader–employee incongruence in humor, employees who benefit from heterogeneous resources show more innovative behavior. In addition, this study also found that the high–high congruence condition had higher innovative behavior than the low–low congruence condition, and more favorable effects on innovative behavior were produced when employees were at a higher level of humor than leaders. This showed that different resource matching has different impact on employee innovation behavior. The results clarified the specific role of leader and employee humor in promoting employee innovative behavior and further expanded the research on antecedents of innovative behavior.

Fourth, this study improved the boundary conditions between leader–employee congruence in humor and innovative behavior by exploring the moderating role of dynamic tenure. On the one hand, there are certain boundary conditions for leader humor to produce positive results (Pundt, 2015). Although previous studies have explored the boundary conditions of employee innovation from the perspective of tenure (Woods et al., 2018) and dynamic tenure (Li et al., 2019), the leader–employee congruence in humor has not been considered. This study regarded dynamic tenure as a kind of human and psychological resources, which enriched the research perspective of dynamic tenure. On the other hand, meta-analyses have found considerable inconsistencies with respect to the tenure–innovation relationship (Ng and Feldman, 2013), with innovation increasing with tenure for some individuals (Li et al., 2019). The results showed that the impact of dynamic tenure on innovation depends on the congruence or incongruence of leader–employee humor. Specifically, this study found that, in the low dynamic tenure group, the incongruence/congruence effect of leader–employee humor has a stronger impact on employee innovation behavior. This means that employees with shorter dynamic tenure can quickly obtain the innovative resources from the new environment. These results not only expanded the boundary conditions between leader–employee congruence in humor and innovative behavior, but also complemented the research on dynamic tenure.

## Limitations and Future Research

This study still has some limitations. First, all the variables are self-reported. Although this study adopted multiple-time-point design and it is reasonable to use self-report method to measure individual intention, there may still be a social approval effect (Marlowe and Crowne, 1961). Future studies should adopt methods to reduce the common method biases, such as paired questionnaire or combining self-evaluation with other’s evaluation. Second, the sample size of this study is small because of the difficulty in collecting paired samples of leaders and employees. Future studies should obtain larger samples to verify the external validity of the research results. Third, this study did not explore the mediation mechanism between leader–employee congruence in humor and innovative behavior. Future studies can explain this mediation mechanism from some theoretical perspectives, such as leader–member exchange (Yam et al., 2018) and positive emotion (Cooper et al., 2018). Fourth, this study discussed the boundary conditions of leader–employee congruence in humor only from the perspective of dynamic tenure. Future studies can expand the boundary conditions from other theoretical perspectives and hierarchical variables, such as autonomy support at the higher unit (Liu et al., 2011) and team learning behavior (Hirst et al., 2011). Fifth, this study does not consider the attributes (positive or negative) of humor, but focused only on the use of humor by leaders or employees. Future researchers can pay more attention to the different effects of different types of humor, such as positive or negative humor (Tremblay, 2017).

### Practical Implications

First, organizations should pay special attention to the importance of humor in the process of stimulating employee innovative behavior. As the “lubricant” of social interaction (Bippus et al., 2011), humor can provide a variety of innovative resources. For example, organizations such as Yahoo and Southwest Airlines have encouraged use of fun and appropriate humor in the workplace to appeal to employees. In addition, presumably as a result of the idea that humor can be used as a management tool, many organizations now provide humor workshops for their employees as a means to improve organizational effectiveness (Wood et al., 2011). This study also found that humor can provide cognitive, emotional, and relational resources for innovation and ultimately promote employee innovative behavior. Therefore, in daily management activities, organizations can strengthen the training of leader humor and employee humor so as to create a positive organizational environment and good resource conditions for innovation. In addition, organizations can take great care in developing recruitment strategies that are aimed at identifying and selecting potential employees who are humorous.

Second, organizations should care about the dual matching between leaders and employees in the process of stimulating humor. This study found that the complementary fit of leader humor and employee humor is more conducive to the generation of innovative behavior. Especially, when employee humor is higher than his or her leader, the employee has stronger intrinsic motivation and more emotional resources for innovative behavior. Therefore, more humor is not always better; managers need to maintain the humor at a moderate level in the organization. In particular, excessive humor may be interpreted as inauthentic humor, which increases organizational cynicism (Dean et al., 1998). Therefore, according to the different level of the leader humor, employees can be transferred appropriately to achieve a better state of complementary fit between leaders and employees. For example, for leaders with low humor, employees with a high level of humor can be allocated to them so as to promote the generation of employee innovative behavior.

Third, organizations should also consider the role of dynamic tenure in the process of stimulating innovative behavior through leader–employee incongruence in humor. The results of this study showed the restrictive effect of dynamic tenure on innovative behavior. The interesting finding is that employees with shorter dynamic tenure are more likely to obtain resources from leader–employee humor incongruence and engage in innovative behavior, because they are more likely to cherish existing resources and invest it in innovative activities. Although a longer dynamic tenure can enhance communication between leaders and employees and promote high-quality exchange relations (Li et al., 2019), for innovation the conflict environment is more conducive to the creativity. Therefore, it is necessary to consider the influence of dynamic tenure as well as the humor matching between leaders and employees. In the organization, a certain degree of job rotation can not only activate the creative thinking of employees, but also provide more innovative resources for employees, thus contributing to the generation of more innovative behavior.

## Data Availability Statement

The raw data supporting the conclusions of this article will be made available by the authors, without undue reservation.

## Ethics Statement

This study was carried out in accordance with the ethical guidelines of the American Psychological Association with written informed consent from all subjects. I introduced the research purpose, goals, and plans to each participant and asked their permission to participate in this research.

## Author Contributions

YY contributed to designing, analyzing, and writing the study.

## Conflict of Interest

The author declares that the research was conducted in the absence of any commercial or financial relationships that could be construed as a potential conflict of interest.
